# Factor VII Deficiency in Systemic Mastocytosis with an Associated Myeloid Neoplasm

**DOI:** 10.3390/hematolrep16010014

**Published:** 2024-03-12

**Authors:** Giorgio Rosati, Sofia Camerlo, Alessandro Fornari, Valerio Marci, Barbara Montaruli, Alessandro Morotti

**Affiliations:** 1 Department of Clinical and Biological Sciences, San Luigi Gonzaga Hospital, University of Turin, 10043 Orbassano, Italy; sofia.camerlo@unito.it (S.C.); alessandro.morotti@unito.it (A.M.); 2 Department of Oncology, Division of Pathology, San Luigi Gonzaga Hospital, 10043 Orbassano, Italy; a.fornari@sanluigi.piemonte.it (A.F.); v.marci@sanluigi.piemonte.it (V.M.); 3 Laboratory Analyses, Azienda Ospedaliera Ordine Mauriziano, 10128 Turin, Italy; bmontaruli@mauriziano.it

**Keywords:** mastocytosis, factor VII deficiency, bleeding disorder, polycythemia vera

## Abstract

Factor VII (FVII) deficiency is a rare bleeding disorder that can be classified as congenital or acquired, and the majority of acquired cases are due to vitamin K deficiency or liver disease. Isolated acquired FVII deficiency is a rare occurrence and has been associated with inhibitors or auto-antibodies. Here, we describe a patient with polycythemia vera who developed systemic mastocytosis and FVII deficiency simultaneously. FVII deficiency was not caused by inhibitors and improved with antineoplastic treatment. Acquired FVII deficiency has been reported in cases of sepsis, possibly due to proteolytic degradation induced by the activation of monocytes or endothelial cells. Malignancies have been shown to cause a depletion in circulating FVII through the direct binding of cancer cells. This case report suggests a potential association between SM associated with a hematological neoplasm (SM-AHN) and acquired FVII deficiency. Further evaluations are recommended in patients with systemic mastocytosis to gain a better understanding of the relationship between pathological mast cells and clotting factor concentrations.

## 1. Introduction

Factor VII (FVII) deficiency is a rare bleeding disorder, classified as congenital or acquired [1].

Most of the acquired cases are due to vitamin K deficiency, liver failure, or consumption syndrome caused by disseminated intravascular coagulation. Therefore, these conditions are associated with defects in other vitamin K-dependent clotting factors. FVII deficiency alone is extremely rare, and it can be caused by the presence of an inhibiting antibody [2,3]. Only a few cases have been reported in the literature [4].

Systemic mastocytosis (SM) is a heterogeneous disease sustained by the clonal proliferation of abnormal mast cells (MC) that infiltrate the bone marrow and/or other extracutaneous organs [5,6].

The World Health Organization has classified SM according to its pathological and clinical characteristics. SM can be either an indolent/smoldering (ISM/SSM) or an advanced disease (AdvSM), which include aggressive SM (ASM), SM associated with a hematological neoplasm (SM-AHN) and mast cell leukemia (MCL) [7].

Here, we present a case of transient acquired factor VII deficiency associated with SM.

## 2. Materials and Methods

This report was conducted according to ethical principles consistent with the Declaration of Helsinki. Since this article used only de-identified patient records and did not involve the collection, use, or transmittal of individually identifiable data, it was exempt from Institutional Review Board approval.

Written informed consent for publication was obtained from the patient.

The histological diagnosis was conducted according to the 2022 World Health Organization’s (WHO) Classification of Haematolymphoid Tumours.

The medical literature was investigated by searching both the MEDLINE and EMBASE databases.

A next-generation sequencing-based analysis of bone marrow specimens was performed using SOPHiA DDM™ Myeloid Solution (by SOPHiA GENETICS™, Rolle, Switzerland).

Flow cytometry analyses were carried out with a Becton Dickinson FACSLyric™ 12 colors (Franklin Lakes, NJ, USA) using lyophilized antibodies from the EuroFlow™ Consortium (Zutphen, The Netherlands). Human genome reference: GRCh37/hg19. Consulted databases: dbSNP v151; ClinVar v20200312; COSMIC v87; dbNSFP v2.9; ESP5400; ExAC r0.3.1; G1000 v5.20130502; GISAID EpiCoV v20200512; GnomaAD r2.1; JAX-CKB v2020082. We did not evaluate intronic, UTR, intergenic and synonymous alterations. Average read depth was equal to or greater than 1000× per nucleotide base.

Factor VII activity was measured in a one-stage coagulation assay (HemosIL Factor VII, Werfen, Bedford, MA, USA) on an ACL TOP automatic blood coagulation analyzer (Bedford, MA, USA).

For immunohistochemistry, the following antibodies were used: polyclonal rabbit anti-human MPO (Dako, Agilent Technologies, Santa Clara, CA, USA); polyclonal rabbit anti-human CD117/c-KIT (Dako); monoclonal mouse anti-human CD34 class II, clone QBEnd/10 ready-to-use (Dako Omnis); monoclonal mouse anti-human CD71, clone Mrq-48 (Cell Marque, Rocklin, CA, USA); monoclonal mouse anti-human Mast Cell Tryptase, clone AA1 (Medac, Wedel, Germany); monoclonal rabbit anti-human CD25, clone EP-218 (Epitomics, Burlingame, CA, USA). All the analyses were performed on the Dako Omnis platform.

## 3. Case Presentation

In March 2020, a 54-year-old Caucasian male attended our clinic due to a diagnosis of polyglobulia. On blood tests, he presented with Hb at 17.3 g/dL, Hct at 55.7%, WBC at 6.46 × 10^3^/μL (neutrophils: 5.23 × 10^3^/μL, lymphocytes: 1.66 × 10^3^/μL, monocytes: 1.36 × 10^3^/μL), platelets at 700 × 10^3^/μL, creatinine at 0.67 mg/dL, AST at 28 U/L, ALT at 21 U/L, bilirubin at 0.7 mg/dL, LDH at 580 U/L, a prothrombin time (PT) ratio of 1.06, an activated partial thromboplastin time (aPTT) ratio of 0.99 and electrolytes in the normal range.

The patient reported experiencing only mild and occasional generalized itching. His past medical history did not reveal any allergies, anaphylactic reactions, episodes of angioedema, bone fractures, or gastroenterological symptoms such as abdominal pain, diarrhea, nausea, or vomiting. His family history was negative for cancer and hematologic disorders.

An abdominal ultrasound (US) showed hepatic steatosis, a spleen diameter measuring 12.4 cm and the presence of renal microlithiasis.

On suspicion of a primary hematological disorder, a bone marrow biopsy was carried out. The biopsy confirmed a profile compatible with a myeloproliferative syndrome, leaning more towards the polycythemia vera (PV) type (the findings were not entirely pathognomonic). Although immunohistochemistry was performed using an antibody against CD117, no cells positive for this marker were identified. Molecular investigations showed positivity for the JAK2-V617F mutation (VAF 15%).

At the time of diagnosis, the patient was 54 years old and had no history of thrombotic events, so he was treated accordingly with low-dose antiplatelet therapy and bloodletting to achieve a target Hct level < 45%. During the following two years, the pruritus improved and the hematocrit remained stable; however, there was a gradual decrease in platelet count, which dropped to 114 × 10^3^/μL. In November 2022, blood tests revealed an increase in GGT (175 U/L) and ALP (640 U/L) values, while the bilirubin remained normal. Subsequently, a bone ALP assay was conducted and indicated an elevated level (115 μg/L). The patient also reported having lost 5 Kg in the preceding months while experiencing increased fatigue. Furthermore, abdominal ultrasonography was repeated, and no changes were observed in either the biliary tract or liver; however, an increase in the size of the spleen was noted. An MRI evaluation of the abdomen confirmed an increased spleen size (bipolar diameter 20 cm) and revealed some hyperintense spots in the bones examined.

Due to the concerning signs of the progression of hematologic disease, it was decided to repeat the bone marrow biopsy, which resulted in a dry tap. Histologic examination revealed bone marrow extensively infiltrated by mast cells with a spindle appearance and immunophenotypic atypia (CD25+, CD117+, Tryptase+) arranged in solid aggregates that accounted for 50–60% of cellularity. In the regions amidst the nodules, the hematopoietic tissue demonstrated a megakaryocyte morphology that was coherent with the known myeloproliferative disorder (MF-1 reticulin pattern). Thus, the observations were consistent with systemic mastocytosis associated with a hematologic malignancy.

Cytofluorometry on peripheral blood showed the presence of less than 0.1% CD117+ and CD25+ cells, and the serum tryptase assay yielded a result of 772 μg/L.

Based on our findings and on the diagnostic criteria of 2022 from the WHO and ICC (International Consensus Classification), a diagnosis of systemic mastocytosis associated with a hematologic neoplasm (SM-AHN) was formulated.

An NGS analysis was also performed on bone marrow to assess the mutational status of 32 genes, showing the presence of mutation in IDH2 (Arg140Gln, VAF 44.9%), SRSF2 (Pro95Arg, VAF 17.5%; Pro95Leu, VAF 31.7%), CBL (His398Gln, VAF 11.7%), JAK2 (Val617Phe, VAF 32.9%) and KIT (Asp816Val, VAF 24.3%). Systematic testing for cancer-predisposing syndromes was not performed due to the patient’s age and lack of suggestive family history.

Simultaneously with the diagnosis of systemic mastocytosis, laboratory tests revealed a PT prolongation with a ratio of 1.80 (checked twice), with normal values of aPTT (ratio of 1.16), fibrinogen levels and liver function tests, including AST/ALT, bilirubin, albumin, cholesterol and triglycerides. No bleeding manifestations were observed, besides minor local bleeding during bone marrow biopsy.

In consideration of the isolated prolongation of PT, we assayed FVII activity and found it to be 38%. To differentiate between factor deficiency and inhibitors against specific clotting factors or lupus anticoagulant (LA) interference, we performed PT on a plasma sample mixed with normal plasma. In this type of test, if the mixture’s PT result is not correct, the patient may have an inhibitor, such as a lupus anticoagulant or inhibitors against specific clotting factors. If there is a correction in the PT result of the mixture, this may be an indication of a coagulation factor deficiency. Following the CLSI guidelines, we conducted factor VII tests on three dilutions of the plasma sample to determine linearity and parallelism. Non-parallelism is a phenomenon associated with LA and pathological inhibitors against specific clotting factors, while parallelism is typical of factor deficiency. The ACL TOP analyzer software enables us to perform factor assays with three dilutions and assess parallelism based on the coefficient of variation (CV). After correcting for the dilution factors, the resulting potencies should be deemed acceptable (parallel) if the deviation from the average is not more than 15%. In our patient, the PT mixture was corrected completely (PT ratio = 1.01), and we obtained a “parallelism” CV of 1.5%, confirming a factor deficiency for this patient. Since the presence of a specific anti FVII inhibitor was excluded, we did not perform a Bethesda assay [8].

Reduced levels of FVII can be caused by various factors, including therapy with anticoagulants such as AVK or DOAC, liver disease, vitamin K deficiency, consumption coagulopathies and the acute phase of a thrombotic event. In this case, the patient was not taking anticoagulants, had not experienced any thrombotic events and showed no clinical or laboratory changes indicative of hemodilution.

Additionally, liver disease, disseminated intravascular coagulation and vitamin K deficiency result in a deficiency in multiple coagulation factors. The patient presented with an elongation of PT, while the PTT, platelet count and liver synthesis values (total proteins, albumin, lipid balance and fibrinogen) were normal. Radiological examinations, such as abdominal ultrasonography and abdominal MRI, showed no signs of liver disease. Thus, a combined factor deficiency was excluded and FVII activity was initially tested, as it is the most commonly associated factor deficiency with isolated PT prolongation.

The patient did not experience any thrombotic events, and as there was no laboratory or clinical evidence of liver failure, FV, FX, and protein C and S assays were not performed.

Based on his hematological diagnosis, age and lack of history of thrombosis, the patient was treated with midostaurin 100 mg BID, and his clinical conditions improved according to the tryptase levels, which became as low as 181 μg/L after 2 months. Strikingly, PT time also decreased, and just after one month of treatment, the FVII levels returned to normal values (73%).

**Figure 1:**
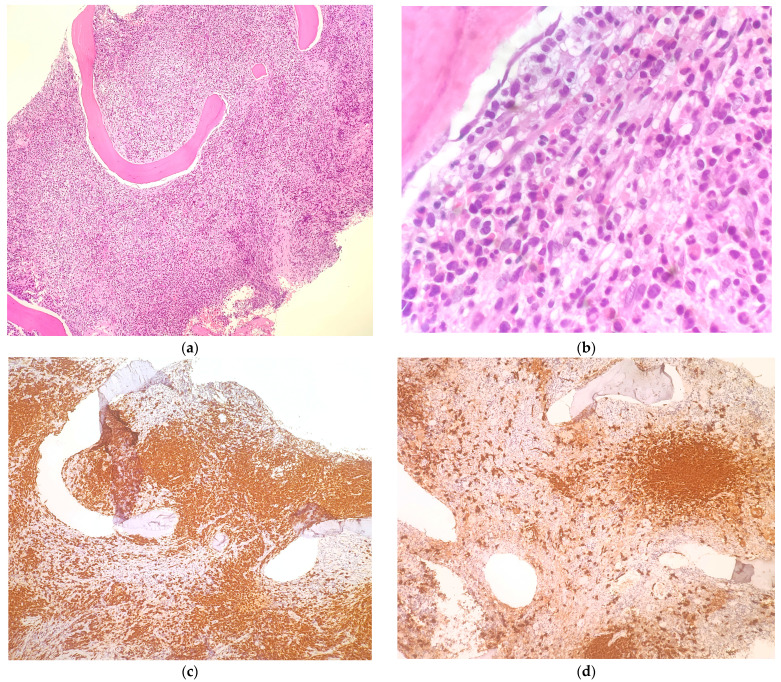
Bone marrow biopsy performed in November 2022 showed extensive bone marrow infiltration by cells with epithelioid and spindle cell morphology ((**a**,**b**) H&E staining with 100× and 400× magnification, respectively). Immunostaining for Mast Cell Tryptase was strongly positive in this cell population ((**c**) 100× magnification). The same immunostaining performed on bone marrow biopsy after 6 months of treatment revealed a consistent reduction in mast cell infiltrate ((**d**) 100× magnification).

At 6 months after the diagnosis, the serum tryptase level decreased to 165 μg/L, and the new bone marrow biopsy showed only some remaining nodular aggregates of mast cells, representing 15% of the total cell population, indicating partial remission (see Table 1 and Figure 1). At this time, the patient was asymptomatic.

## 4. Discussion

Neoplastic diseases may be associated with various disorders of the hemostatic process. In particular, the most frequent manifestations are alterations in platelet count (thrombocytopenia or thrombocytosis) and disseminated intravascular coagulation. Thus, clinically, tumors may be associated with both hemorrhagic and thrombotic states.

However, it is important to remember that there are also acquired defects of coagulation factors, which are rare conditions, especially if we consider isolated acquired deficiencies. Acquired hemophilia A and acquired Von Willebrand disease represent the most frequent isolated defects associated with tumors. They are characterized by mild to moderately severe mucocutaneous hemorrhages and excessive bleeding following trauma or surgery, and are accompanied by a prolonged activated partial thromboplastin time [9,10].

In our clinical case, the aPTT was found to be normal, whereas the patient had a prolonged PT and low FVII levels with no past history or current manifestations of bleeding.

The isolated prolongation of PT can be caused by a FVII deficiency. Among the congenital disorders, FVII deficiency is the most common, and FVII levels do not correlate with clinical bleeding manifestations, but only with the minor prolongation of PT [11].

In contrast to congenital FVII deficiency, acquired FVII deficiency is extremely rare and only few cases are reported in the literature. In particular, it was reported during sepsis, as a potential consequence of proteolytic degradation induced by the activation of monocytes and endothelial cells [12,13]. Other cases associated with major trauma and bone marrow transplantation have been described; however, the mechanisms are not fully understood [14,15].

Acquired FVII deficiency was also associated with neoplastic disorders such as pleural liposarcoma, Wilms tumor, atrial myxoma and some hematological tumors, including multiple myeloma, acute myeloid leukemia and lymphoma. Nevertheless, these conditions were mostly linked to the presence of inhibitors [13,16,17,18,19,20,21,22].

On the other hand, in various published works, SM has been related to bleeding manifestations, such as mucocutaneous hemorrhage and prolonged bleeding after biopsies, and abnormalities in coagulation tests in about 50% of cases. In 2015, French authors published the results of a national survey on 14 patients with mastocytosis and hemostasis disorders. Four patients had confirmed or strongly suspected primary hemostasis disorders (including von Willebrand disease in two cases), while the remaining 10 patients had secondary hemostasis disorders, which were more often due to multiple factorial deficits. Interestingly, FVII was reduced in eight patients, and alterations in secondary hemostasis were more pronounced during the intense degranulation crisis. It is also noteworthy that coagulation values were corrected solely by steroid administration and not by fresh-frozen plasma, transfusions or vitamin K [23,24,25].

Although the mechanisms remain unclear, some mast cell degranulation products, especially tryptase, heparin and histamine, may have an anticoagulant effect. In 2011, Seidel et al. analyzed a cohort of 68 individuals affected by mast cell activation syndrome (MCAS). They found increased levels of endogenous heparin and altered concentrations of molecules involved in the process of fibrinolysis, such as tPA and PAI [26].

Moreover, in 2008, Sucker et al. reported a case of fatal bleeding in a 37-year-old woman with SM. The authors observed an unexplained prolongation of PTT that was corrected with protamine and steroid administration. Therefore, they concluded that the alteration was likely due to the presence of heparin-like molecules released by pathological mastocytes. In the same year, Koenig and colleagues described a similar case in a young 39-year-old male who was then diagnosed with an aggressive form of mastocytosis [27,28].

In our case, the patient had normal aPTT and prolonged PT with an FVII deficiency. The PT was corrected after the addition of normal plasma, and we obtained a coefficient of variation of 1.5% with dilutions, so an underlying inhibitor-mediated mechanism was ruled out. In addition, simultaneously with the response to therapy, we observed the normalization of PT and FVII levels. Therefore, we can conclude that the patient had an acquired FVII deficiency related to SM that completely regressed upon treating the underlying neoplastic cause.

## 5. Conclusions

In several publications, mastocytosis has been associated with hemorrhagic phenomena and abnormalities in coagulation tests. Although in most cases, isolated PT prolongation in a neoplastic context is related to the presence of inhibitors, here, we present a case in which the alteration was assayed and found to be independent of the presence of autoantibodies. In addition, both PT and FVII activity promptly normalized shortly after treatment was initiated, highlighting the close correlation between laboratory changes and disease activity itself.

While the association between mastocytosis and clinical or laboratory hemorrhagic tendency has been described, there is still a gap in the literature regarding both the systematic approach to this issue and the biological mechanisms underlying these observations.

This case report points to a potential link between SM-AHM and acquired FVII deficiency. Thus, we suggest that further evaluations are advisable in patients with SM to better understand the link between pathological mastocytes and clotting factor concentrations.

## Figures and Tables

**Table 1:** The analyses performed on the peripheral blood and the bone marrow at diagnosis.

	March 2020 (Diagnosis of PV)	November 2022 (Diagnosis of SM)	6th Month from Diagnosis of SM
WBC × 10^3^/μL	6.46	6.03	5.20
Hb (g/dL)	17.3	12.1	12.7
Hct (%)	55.7	39.1	40.5
PLTs × 10^3^/μL	700	114	203
PT ratio	1.06	1.80	1.16
PTT ratio	0.99	1.16	1.13
Fibrinogen (mg/dL)	330	440	436
AST (U/L)	28	19	27
ALT (U/L)	21	9	19
GGT (U/L)	46	175	37
ALP (U/L)	107	640	110
Total bilirubin (mg/dL)	0.70	1.10	0.80
Tryptase (μg/L)	–	772	165
Factor VII activity (%)	–	38	70
Spleen diameter (cm)	12.5	20	19
Bone marrow	Marked expansion of the megakaryocytic lineage. Large MK with hyperlobulated nuclei that form small, loose aggregates. MF-0.	50–60% of cellularity consists of confluent aggregates of CD117+ and CD25+ mast cells. MK morphology compatible with the known MPN. MF-1.	Trilineage hyperplasia. MK with a tendency to form loose aggregates. 15% of the cellularity is composed of a small number of nodular aggregates of CD25+ and CD30+ mast cells. MF-2.

MK, megakariocytes; MF, grading of bone marrow fibrosis according to WHO.

## Data Availability

No new data were created or analyzed in this study. Data sharing is not applicable to this article.
